# Does fertilization explain the extraordinary hydraulic behaviour of apple trees?

**DOI:** 10.1093/jxb/erz070

**Published:** 2019-02-22

**Authors:** Barbara Beikircher, Adriano Losso, Marilena Gemassmer, Steven Jansen, Stefan Mayr

**Affiliations:** 1 University of Innsbruck, Institute of Botany, Sternwartestrasse, Innsbruck, Austria; 2 Ulm University, Institute of Systematic Botany and Ecology, Albert-Einstein-Allee, Ulm, Germany

**Keywords:** Drought tolerance, embolism, hydraulic conductivity, pit anatomy, stomatal closure, stomatal conductance, turgor, water potential, xylem anatomy

## Abstract

Fertilization of woody plants plays a central role in agriculture and forestry, but little is known about how plant water relations are thereby affected. Here we investigated the impact of fertilization on tree hydraulics, and xylem and pit anatomy in the high-yield apple cultivars Golden and Red Delicious. In fertilized trees of Golden Delicious, specific hydraulic conductivity of branch xylem, hydraulic conductance of the root system, and maximum stomatal conductance increased considerably. In Red Delicious, differences between fertilized and control trees were less pronounced. In both cultivars, xylem embolism resistance of fertilized trees was significantly lower and stomatal closure occurred at lower water potentials. Furthermore, water potential at turgor loss point and osmotic potential at full saturation were higher and cell wall elasticity was lower in fertilized plants, suggesting reduced drought tolerance of leaves. Anatomical differences were observed regarding conduit diameters, cell wall reinforcement, pit membrane thickness, pit chamber depth, and stomatal pore length, with more pronounced differences in Golden Delicious. The findings reveal altered hydraulic behaviour in both apple cultivars upon fertilization. The increased vulnerability to hydraulic failure might pose a considerable risk for apple productivity under a changing climate, which should be considered for future cultivation and management practices.

## Teaser

Fertilization of high-yield apple cultivars leads to increases in hydraulic efficiency and decreases in embolism resistance, as well as drought tolerance of leaves, whereby the influence is highly cultivar specific.

## Introduction

In commercial orchards, high-yield cultivars are grown under optimized irrigation and fertilization regimes to achieve the best results in terms of quality and productivity (Jones, 1992; Naor and Girona, 2012; Beikircher *et al.*, 2013). In a previous study, Beikircher *et al.* (2013) reported that under these conditions, trees can exhibit an extraordinary hydraulic behaviour. Measurements on three economically important apple cultivars revealed an adjustment of hydraulic traits in order to optimize gas exchange and thus productivity—even at the risk of hydraulic failure: 50% loss of hydraulic conductivity in leaves and branches, turgor loss point, and initial damage to mesophyll cells occurred around or even at less negative water potentials than full stomatal closure (Beikircher *et al.*, 2013). Such a late stomatal closure poses a considerable risk for plant survival, and the authors suggested that this striking behaviour is based on the selection of high-yield cultivars and optimized water supply. High-yield apple cultivars are artificial plant systems, with scions, bred for high quantity and quality, grafted on selected rootstocks, which in turn strongly control tree vigour and fruit characteristics. As for water supply, daily irrigation of the orchards ensured optimal water supply, with constant soil water potentials around –0.2 MPa. In addition to the nature of the plant material and daily irrigation, it is possible that fertilization plays a role in the unusual hydraulic behaviour of apple cultivars.

For the most part, fertilization with plant-available nitrogen (N) increases growth rates and leaf area (Cooke *et al.*, 2005; Pallardy, 2008), which in turn require significant anatomical and hydraulic adjustments to maintain sufficient water supply to the whole plant. Accordingly, in several species, an increase in specific hydraulic conductivity (*k*_s_) of stems or branches was observed upon fertilization (e.g. Bucci *et al.*, 2006; Hacke *et al.*, 2010; Plavcová and Hacke, 2012; Plavcová *et al.*, 2013; Villagra *et al.*, 2013; Medeiros *et al.*, 2016). However, positive effects of N on growth and *k*_s_ are not universal (Clearwater and Meinzer, 2001; Villagra *et al.*, 2013; Faustino *et al.*, 2015; Medeiros *et al.*, 2016; Wang *et al.*, 2016). Also, as indicated by a similar or even reduced leaf-specific hydraulic conductivity (*k*_l_), the increase in *k*_s_ was often not sufficient to compensate for the higher leaf area in fertilized plants (Ewers *et al.*, 2000; Clearwater and Meinzer, 2001; Bucci *et al.*, 2006; Domec *et al.*, 2009; Hacke *et al.*, 2010; Faustino *et al.*, 2013). This in turn bears consequences for other hydraulic parameters as, according to the Ohm’s law analogy (Tyree and Ewers, 1991; Hubbard *et al.*, 2001), *k*_l_ is tightly coupled with leaf water potential (Ψ_l_) and, in consequence, stomatal conductance (*g*_s_). In several studies, no effect (Clearwater and Meinzer, 2001) or a decrease in Ψ_l_ and/or *g*_s_ (Ewers *et al.*, 2000, Bucci *et al.*, 2006, Domec *et al.*, 2009) was observed upon fertilization. Besides N, fertilization with phosphorus (P) and potassium (K) was also found to alter hydraulic conductivity, although effects were often less pronounced (Harvey and Driessche, 1997; Bucci *et al.*, 2006; Faustino *et al.*, 2013; Medeiros *et al.*, 2016). In the case of K though, changes are related to an ionic effect (e.g. Nardini *et al.*, 2011; Oddo *et al.*, 2011) rather than to structural changes (see below).

From an anatomical point of view, the observed increase in hydraulic efficiency upon fertilization in some studies was related to an increase in conduit diameter (e.g. Hacke *et al.*, 2010; Plavcová and Hacke, 2012; Plavcová *et al.*, 2013). This is not surprising because conductivity is proportional to the fourth power of conduit diameter (Tyree and Zimmermann, 2002). Also, intervessel pit characteristics affect hydraulic efficiency (e.g. Choat *et al.*, 2008; Lens *et al.*, 2011), but potential changes of bordered pits due to fertilization are unclear. Conduit and pit anatomy may also influence a trees’ resistance to xylem embolism. Bordered pit characteristics such as pit chamber depth, pit aperture area, pit border area, and pit membrane ultrastructure are known to affect air-seeding, although this process is only partly understood (Sperry and Tyree, 1988; Lens *et al.*, 2011; Jansen and Schenk, 2015). In particular, pit membrane thickness was found to be highly variable and suggested to be an important determinant of resistance against drought-induced embolism (Jansen *et al.*, 2009; Li *et al.*, 2016). Furthermore, embolism resistance has also been reported to correlate with the mechanical strength of conduit walls; that is, the thickness to span ratio [(*t*/*b*)_h_^2^; Hacke *et al.*, 2001; Lens *et al.*, 2011, 2013].

There are less than a dozen studies investigating changes in embolism resistance upon fertilization, and reported findings are contradictory (see Supplementary Table S1 at *JXB* online for a synthesis). In hybrid poplar, embolism resistance has been found to decrease upon N fertilization, slightly increase with P fertilization, and to be unaffected by K (Harvey and Driessche, 1997, 1999; Hacke *et al.*, 2010; Plavcová and Hacke, 2012; Plavcová *et al.*, 2013). Vice versa, in tropical trees, an increase in xylem embolism resistance has been observed in N-fertilized trees, but no effect was found in trees fertilized with P or K (Bucci *et al.*, 2006; Villagra *et al.*, 2013). Also, upon an elaborated fertilization regime with P, K, Ca, and Mg, Ewers *et al.* (2000) observed an increase in embolism resistance of roots, but no effect on branches of *Pinus taeda*.

It is clear that fertilization does impact tree hydraulics, but how, and to what extent, appears to be highly variable. The effect on plant productivity and hydraulics depends on various factors such as plant species (e.g. Villagra *et al.*, 2013; Medeiros *et al.*, 2016), fertilizer (type and amount), growing conditions (e.g. water supply, light), plant age, and experimental design (e.g. Faustino *et al.*, 2015; Wang *et al.*, 2016). Moreover, since different outcomes have been observed even under similar controlled conditions for a single species, the effect of fertilization on plant water relations might be highly dynamic (Hacke *et al.*, 2010). Many studies were done on pine species or hybrid poplar, with the main focus on hydraulic efficiency and gas exchange. Little is known about embolism resistance of different organs, root and leaf hydraulics, stomatal control and hydraulic safety margins, and xylem anatomy including pit properties and stomata.

Our study was aimed at analysing the impact of fertilization on hydraulic and underlying anatomical traits of two high-yield apple cultivars, in order to scrutinize whether it might explain their extraordinary hydraulic behaviour. Analyses were carried out on Red Delicious, which in a previous study showed low embolism resistance and a remarkably late stomatal closure, and the more drought-tolerant Golden Delicious (Beikircher *et al.*, 2013). We studied hydraulic parameters of different organs of fertilized and control trees, and correlated them with various anatomical traits. Based on well-known positive fertilization effects on growth and productivity of apple trees (Jackson, 2003), we hypothesized that fertilization leads to increased hydraulic efficiency, but also, due to associated anatomical changes, to reduced hydraulic safety of high-yield apple cultivars. As a result, we expected adjustments to occur at different levels (cell to whole organs) and to be more pronounced in Red Delicious.

## Materials and methods

### Plant material, experimental design, and study site

Measurements were made on the *Malus domestica* Borkh. cultivars ‘Golden Delicious’ and ‘Red Delicious’ grafted on M9 rootstocks. The general experimental design was based on management practices in commercial orchards, but only half of the plants were fertilized and plants were not irrigated. For a list of acronyms with respective definitions and units used in the following, see Supplementary Table S2.

In April 2014, a total of 60 Golden and Red Delicious trees were planted in a south-exposed test field in Innsbruck (600 m a.s.l.; 47°16'2''N, 11°23'34''E); cultivars were randomly distributed in six rows. The distance between trees within a row was 1 m, while the distance between tree rows was 1.5 m. In the course of planting, in three randomly chosen rows, 15 g of organic nitrogen fertilizer (Ikosan, 13–14% N) per tree were worked into the soil (fertilized trees). Trees in the remaining rows were not fertilized (control trees). Plants were watered every second day throughout the 2014 growing season to ensure optimal plant establishment, but in the following years plants were not irrigated. In spring 2015 and 2016, on an area of ~50×50 cm around trees chosen for fertilization treatment, 15 g of an inorganic NPK fertilizer (Compo, Novatec, 14%N) per tree was applied. A wash-out of nutrients could be excluded because (i) the fertilizer was directly worked into the soil; (ii) there was a distance of at least 75 cm to the neighbouring row; and (iii) no height level difference existed between rows. Furthermore, in a previous study on the same cultivars on the same rootstock but older trees, the main rooting area was within 30 cm from the stem. Thus, the distance between the rows was assumed to be sufficient to avoid roots of control trees reaching fertilized soil. This assumption is confirmed by our observation that within control trees no effect of the position in the test field (i.e. neighboured by control or fertilized trees) on growth was observed. In all years, trees were pruned in spring and all but 10 flowers per tree were removed to avoid reduced shoot growth due to high allocation of resources in fruits. The main study year 2015 was characterized by an exceptionally warm and dry summer, with a precipitation and temperature deviation of –24% and +3.2 °C from the long-term mean in July (127 mm, 18.5 °C) and a deviation of –20% and +2.1 °C from the long-term mean in August (122 mm, 17.8 °C), respectively (https://zamg.ac.at).

### Sampling procedure

For measurements carried out in the laboratory, branches of randomly selected trees were harvested from mid August to mid September 2015. Harvesting and sampling were done according to the protocol by Beikircher and Mayr (2016) to maintain the native hydraulic state of shoots *in planta*. In short, branches were cut at the base, immediately re-cut several centimetres under water, wrapped in dark nylon bags with wet paper towels, and transported to the laboratory, where they were stored in a cold temperature chamber (5 °C) until measurements were conducted.

### Tree growth

At the end of the 2015 growing season, current year tree top and shoot length was determined with a measuring tape on all trees. Furthermore, the width of the xylem ring developed in the past season was measured on transverse sections made for xylem anatomical analyses (see ‘Xylem anatomy’). Ring width was measured at five different positions across the section, and averaged values per sample were used to calculate means per cultivar and treatment.

### Pressure–volume analyses

For pressure–volume analyses, 10 randomly selected leaves of five saturated branches per cultivar and treatment were cut, and turgid weight (TW) was measured with an analytical balance (Sartorius BP61S, 0.0001 g precision, Sartorius AG, Göttingen, Germany). Leaves were then dehydrated on the bench and, at intervals, FW was measured before and after analysing the respective leaf water potential (Ψ_l_; MPa) with a pressure chamber (Model 1000 Pressure Chamber, PMS Instrument Company, Corvallis, OR, USA). Leaves were then oven-dried at 80 °C for 24 h to obtain the DW, and the relative water saturation deficiency (WSD) was calculated.

For each single leaf, 1/Ψ_l_ was plotted versus WSD. The turgescent section of the curve was fitted with a parabolic function and the osmotic section with a linear regression (see Tyree and Hammel, 1972) using Fig.P 2006 (Fig.P Software Inc., ON, Canada). The osmotic potential at full saturation (Ψ_osat_; MPa) was determined as the intersection of the linear regression with the *y*-axis, the water potential at turgor loss point (Ψ_TLP_; MPa) from the intersection of the parabolic and the linear regression function, and cell wall elasticity (*a*_ela_) was estimated by the opening width of the parabola (the lower the *a*_ela_, the more elastic the tissue; see Ganthaler and Mayr, 2015). Values of single leaves were then averaged per cultivar and treatment, respectively.

### Vulnerability analyses

Embolism resistance was analysed on up to five shoots per cultivar and treatment developed in the 2014 and 2015 growing seasons. Analyses were done with the Cavitron method (Cochard, 2002; Cochard *et al.*, 2005) using a 28 cm custom-built rotor. The use of 2-year-old shoots was necessary as younger shoots were too short for the 28 cm rotor, which was required to avoid open vessel artefacts (Choat *et al.*, 2010; Cochard *et al.*, 2010; Torres-Ruiz *et al.*, 2014; for information on vessel length in the study cultivars, see Beikircher and Mayr, 2016). Samples were cut from branches under water by re-cutting branches repeatedly from both sides with a pruning knife. About 5 cm of both sample ends were debarked and sample ends finally trimmed with a sharp wood carving knife (see Beikircher and Mayr, 2016) before fixing them in the cuvettes of the rotor.

Cuvettes were then filled with distilled and filtered (0.22 µm) water containing 0.005% (v/v) ‘Micropur Forte MF 100F’ (Katadyn Products Inc., Wallisellen, Switzerland) to prevent microbial growth (Beikircher and Mayr, 2008), and rotational speed was set to a target xylem pressure (P) of –0.25 MPa. After an equilibration time of ~10 min, the water flow from the upstream to the downstream reservoir was measured and hydraulic conductance calculated (for details, see Beikircher *et al.*, 2010). Hydraulic conductance was then repeatedly determined at gradually increased rotational speed (and thus decreased P) until flow was no longer measurable. Percentage loss of hydraulic conductivity (PLC) was calculated as the ratio of the actual and the first (i.e. maximum) measured hydraulic conductance value and plotted versus the respective xylem pressure. Curves were fitted using an exponential sigmoidal equation (Equation 1) given in Pammenter and Vander Willigen (1998):

$$\begin{array}{l} PLC=100/\left\{1+exp\left[a\left(PP_{50}\right)\right]\right\} \end{array}$$ (1)

where P is the corresponding xylem pressure (MPa), *a* is related to the slope of the curve, and P_50_ is the pressure value corresponding to 50% loss of conductivity. Additionally, the values of P at the onset (P at 12%; P_12_) and at full embolism (P at 88%; P_88_) were calculated (Beikircher *et al.*, 2010, 2013).

### Hydraulic conductance of the branch xylem and the root system

The specific hydraulic conductivity (*k*_s_; cm^2^ s^–1^ MPa^–1^) of the branch xylem was measured in summer 2015 and 2016 on 8–10 shoots developed in the respective season per cultivar and treatment. Up to 8 cm long samples were cut from shoots under water, debarked, and sample ends accurately trimmed with a wood carving knife. Samples were then connected to a Xyl’em apparatus (Bronkhorst, Montigny les Cormeilles, France) and perfused with the same solution as used for vulnerability analyses. Hydraulic conductance was measured at 4.5 kPa and normalized by xylem area and sample length to obtain *k*_s_. Samples were flushed at 95 kPa for 20 min and *k*_s_ re-measured to test for native or artefactual embolism due to sample preparation.

Root hydraulic conductance was measured in September 2016 on three trees per cultivar and treatment, using a high-pressure flow meter (HPFM Gen 3; Dynamax, Houston, TX, USA). The stem base was tightly sealed with plastic bags and the obtained receptacle filled with water. Stems were then cut under water ~10 cm above the graft (i.e. ~30 cm above the soil surface). The upper end of the tree stump was debarked over ~8 cm, connected to the HPFM flow meter, and perfused with the same solution used for branch conductivity and vulnerability analyses (see above). Immediately afterwards, 3–5 transient measurements (see Tyree *et al.*, 1995) were made and the hydraulic conductance of the root system calculated (*K*_R_; kg s^–1^ MPa^–1^).

### Stomatal behaviour

Stomatal behaviour was measured directly in the field on five branches per cultivar and per treatment on a sunny day in August 2015. To prevent incomplete stomatal opening due to possible drought stress, trees were irrigated the previous evening. First, stomatal conductance (*g*_s_; mmol m^–2^ s^–1^) and Ψ_l_ were repeatedly measured on randomly chosen leaves using a steady-state leaf porometer (SC-1, Decagon Devices, Pullman, WA, USA) and a pressure chamber (see above), respectively. When *g*_s_ reached constant values [~12.00 h central European time (CET)], branches were cut and measurements repeated at intervals until complete stomatal closure. *g*_s_ was measured first, and the respective leaf was then cut to determine Ψ_l_. During dehydration, cut branches were exposed to similar conditions to branches attached to the tree, and measurements were made within a 2 h period to ensure that stomatal closure occurred in response to decreasing Ψ_l_ (Hubbard *et al.*, 2001; Beikircher and Mayr, 2009). Percentage stomatal conductance was then calculated as the ratio of actual and maximal *g*_s_, and plotted against Ψ_l_. Curve fitting was done according to xylem vulnerability analyses (Equation 1), whereby PLC was substituted by percentage stomatal conductance and P_50_ corresponds to Ψ_l_ at 50% *g*_s_. The water potential at full stomatal closure (Ψ_sc_) was defined as the value of Ψ_l_ at 12% *g*_s_ and that at onset of stomatal closure as the value of Ψ_l_ at 88% *g*_s_ (Beikircher *et al.*, 2013).

### Diurnal courses and maximum stomatal conductance

Diurnal courses of Ψ_l_ and *g*_s_ were measured on a sunny day at the end of August 2015. From 05.00 h to 18.00 h CET, 5–8 trees per cultivar and treatment were chosen randomly for measurements of Ψ_l_ and *g*_s_ (see above) at ~2 h intervals. In addition to hydraulic measurements, air temperature (°C), air humidity (%), and global radiation (W m^–1^) were measured with a meteorological station (uEMSet99) and soil water potential with a gypsum block sensor (MicroLog SP; all sensors and datalogger from EMS, Brno, Czech Republic). Climatic measurements were made at 1 min intervals, and 30 min means were calculated for analyses.

Maximum operating stomatal conductance (*g*_smax_) for a given cultivar and treatment was calculated from the eight highest *g*_s_ values measured in the daily course and the eight highest values of the *in situ* analyses of stomatal behaviour (see the respective paragraph above). To obtain Ψ_lmin_, the eight lowest values measured in the daily course (but independent of time) were averaged per cultivar and treatment.

### Xylem anatomy

Anatomy of the branch xylem was analysed on eight shoots per cultivar and per treatment. The samples, which were previously used for hydraulic conductivity analyses, were soaked in an ethanol–glycerol–water solution (1:1:1, v/v/v) for several weeks before cutting transverse sections with a microtome (Sledge Microtome G.S.L. 1, Schenkung Dapples, Zurich, Switzerland). Transverse sections were stained with Etzold’s staining solution, and anatomical parameters were analysed with a light microscope (Olympus BX 41, System Microscope, Olympus Austria, Vienna, Austria) interfaced with a digital microscope camera (ProgRes CT3, Jenoptik, Jena, Germany) and image analysis software [ImageJ, 1.37, National Institutes of Health (NIH), Bethesda, MD, USA, public domain]. In radial sections, individual lumen areas (*A*) were measured directly and the respective diameters (*d*) calculated assuming a circular conduit shape. The mean hydraulic diameter (*d*_h_; µm) was calculated from individual diameters according to Hacke *et al.* (2004); Equation 2:

$$\begin{array}{l} d_{h}=\sum d^{5}/\sum d^{4} \end{array}$$ (2)

Conduit wall reinforcement (*t*/*b*)_h_^2^ was analysed according to Hacke *et al.* (2001) by directly measuring the double wall thickness (*t*) between adjacent conduits and the respective conduit diameter (*b*).

To avoid possible unbalanced statistical weighting of samples with larger numbers of conduits, the mean diameter (*d*_mean_; µm) as well as *d*_h_ and (*t*/*b*)_h_^2^ were first averaged per sample and then means per cultivar and treatment were calculated from averages (Beikircher *et al.*, 2013).

Pit analyses were made on shoots developed in spring 2016 using TEM. In July 2016, three shoots per cultivar and treatment were chosen randomly, harvested according to the protocol given above, and transported to the laboratory. Shoots were cut into 3 mm^3^ blocks in phosphate-buffered saline (PBS) and fixed with 2.5% glutaraldehyde, buffered with 0.1 M phosphate buffer at pH 7.3. After washing with the latter, samples were post-fixed in 2% osmium tetroxide (OsO_4_) which was then removed by washing with a graded ethanol series (30, 50, 70, and 90%). Finally, samples were block-stained with uranyl acetate before embedding in Epon™. Upon polymerization, ultrathin transverse sections were cut with an ultramicrotome (Ultracut, Reichert-Jung, Austria) and observed with a Jeol JEM-1400 transmission electron microscope (Jeol Germany GmbH, Fresing, Germany). On intervessel pits, pit membrane thickness (*T*_m_; nm) and depth of the pit chamber (*L*_p_; nm; see Fig. 2) were measured using the image analysis software ImageJ (see above). *T*_m_ was measured in the centre and at both sides near the annulus, and for *L*_p_ the distances from the non-aspirated membrane surface to the inner edges of the pit apertures were measured. In the latter two cases, averaged values per pit were used to calculate means per cultivar and treatment.

### Leaf anatomy

For leaf anatomical analyses, 10 leaves per cultivar and treatment were chosen randomly, harvested, and saturated in distilled water overnight. Trichomes on the adaxial surfaces were removed with adhesive tape and a coat of clear nail varnish was applied on intercostal areas. Upon drying, the nail varnish was peeled off with adhesive tape, placed on a microscope slide, and analysed with a light microscope interfaced with a digital microscope camera and image analysis software (see ‘Xylem anatomy’). To determine stomatal density (SD; number per mm^2^), stomata were counted on defined areas. Stomatal pore length (*l*; µm) was measured directly on a total of up to 100 stomata. As leaves were kept well watered and under dark conditions until preparation of samples, it can be assumed that stomata were closed (Beikircher *et al.*, 2013). Similar to xylem anatomical parameters, mean values per cultivar and treatment were calculated from values averaged per leaf.

### Statistics

Depending on normal distribution and variance (tested with the Kolmogorov–Smirnov and the Levene test), the following tests were applied to detect significant differences between fertilized and control trees within a cultivar: P_12_, P_55_, P_88_, parameter *a*, Ψ_sc_, *g*_smax_, *K*_R_, year ring width, *d*_mean_, *d*_h_, *d*_max_, and (*t*/*b*)_h_^2^ were tested with the Welch test. All other parameters were analysed with the Student’s *t*-test. All tests were made pairwise at a probability level of 5% using SPSS version 21.

## Results

### Tree growth

Fertilized trees of Golden Delicious had ~5 cm longer current year tree tops and shoots than control trees (Table 1). In Red Delicious, differences were smaller and amounted to ~2 cm. In Golden Delicious, the year ring width was significantly higher in fertilized plants (615±34 µm) than in the controls (502±36 µm). In Red Delicious, the overall increment was about half that of Golden Delicious, and similar between fertilized and control trees.

**Table 1.** Current year length increment of tree tops and side shoots and year ring width of control and fertilized plants of Golden and Red Delicious

	Golden control	Golden fertilized	Red control	Red fertilized
Tree top (cm)	31.75±2.57	36.56±4.00	20.50±2.78	22.62±3.92
Shoots (cm)	19.73±1.20	24.69±1.20*	18.50±1.13	20.27±1.73
Year ring width (µm)	502±36	615±34*	316±19	327±44

Mean s±SE. Asterisks indicate significant differences within a cultivar.

### Hydraulic efficiency, xylem embolism resistance, and stomatal closure

Fertilization resulted in a significant increase in *k*_s_ of the branch xylem (Table 2). In Golden Delicious, *K*_R_ also strongly increased. However, due to the high variation in *K*_R_ (values ranging from 9×10^–6^ kg s^–1^ MPa^–1^ to 5×10^–7^ kg s^–1^ MPa^–1^) between trees, differences were not significant. In contrast, in Red Delicious, a slight, non-significant decrease in *K*_R_ upon fertilization was observed.

**Table 2.** Hydraulic and anatomical parameters of control and fertilized plants of the apple cultivars Golden and Red Delicious

Organ	Parameter	Golden control	Golden fertilized	Red control	Red fertilized
Leaf	Ψ_TLP_ (MPa)	–2.97±0.09	–2.65±0.08*	–2.37±0.05	–2.43±0.11
	Ψ_osat_ (MPa)	–2.56±0.09	–2.39±0.06	–2.14±0.05	–2.21±0.08
	*a*_ela_	1.38±0.27	1.63±0.24	0.96±0.19	1.44±0.24
	*g*_smax_ (mmol m^–2^ s^–1^)	497±44	617±30*	540±30	571±54
	Ψ_sc_ (MPa)	–3.75±0.17	–4.41±0.21*	–4.19±0.23	–4.88±0.26*
	Ψ_lmin_ (MPa)	–2.73±0.11	–3.04±0.07*	–2.52±0.08	–2.55±0.05
	SD (no mm^–2^)	265±17	267±22	253±9	249±11
	*l* (µm)	15.03±0.59	17.61±0.47*	17.27±0.51	15.33±0.36
Branch	*k*_s_ (cm^2^ s^–1^ MPa^–1^)	6.36±0.45	10.48±1.36*	4.74±0.40	7.70±1.09*
	P_12_ (MPa)	–2.90±0.22	–1.16±0.20*	–2.85±0.17	–2.12±0.17*
	P_50_ (MPa)	–5.00±0.07	–3.65±0.07*	–4.77±0.06	–4.29±0.06*
	P_88_ (MPa)	–7.11±0.07	–6.14±0.06*	–6.69±0.06	–6.46±0.05*
	Parameter *a*	0.95±0.06	0.80±0.04	1.04±0.06	0.92±0.05
	*d*_mean_ (µm)	22.00±0.74	25.15±1.83	19.78±0.61	20.71±2.37
	*d*_h_ (µm)	24.52±0.95	26.86±1.91	22.33±0.57	23.18±2.56
	d_max_ (µm)	30.80±1.00	32.89±2.62	27.93±0.58	28.45±3.24
	(*t*/*b*)_h_^2^×10^–3^	5.44±0.40	4.01±0.31*	5.80±0.24	4.10±1.00
	*T*_m_ (nm)	459±50	415±17	382±31	320±40
	*L*_p_ (nm)	367±24	516±38*	318±38	342±23
root	*K*_R_ (kg s^–1^ MPa^–1^×10^–6^)	0.48±0.09	5.00±2.46	0.82±0.25	0.51±0.22

Water potential at turgor loss point (Ψ_TLP_), osmotic potential at full saturation (Ψ_osat_), cell wall elasticity (*a*_ela_), maximum operating stomatal conductance (*g*_smax_), water potential at stomatal closure (Ψ_sc_), minimum leaf water potential (Ψ_lmin_), stomatal density (SD), stomatal pore length (*l*), specific hydraulic conductivity (*k*_s_), xylem pressure inducing 12, 50, and 88% loss of hydraulic conductivity (P_12_, P_50_, P_88_), slope of the vulnerability curve (*a*), mean, hydraulic, and maximum conduit diameter (*d*_mean_, *d*_h_, *d*_max_), cell wall reinforcement [(*t*/*b*)_h_^2^], pit membrane thickness (*T*_m_), pit chamber depth (*L*_p_), and whole root conductance (*K*_R_). Means ±SE. Asterisks indicate significant differences within a cultivar.

**Fig. 1.**
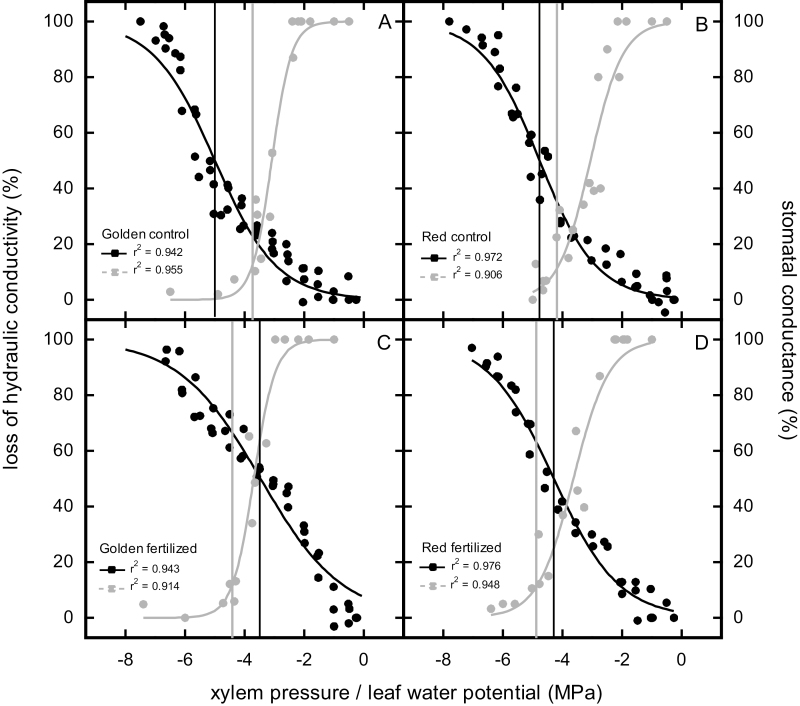
Percentage loss of hydraulic conductivity (black) and percentage stomatal conductance (grey) versus xylem pressure and leaf water potential, respectively, of control (A, B) and fertilized (C, D) plants of Golden Delicious (Golden) and Red Delicious (Red). Vertical lines show xylem pressure at 50% loss of hydraulic conductivity (P_50_; black) and leaf water potential at full stomatal closure (Ψ_sc_; grey), respectively.

Embolism resistance of the branch xylem was significantly decreased upon fertilization in both cultivars. Water potential at 50% loss of hydraulic conductivity (P_50_) decreased from –5.00 MPa and –4.77 MPa in control trees to –3.65 MPa and –4.29 MPa in fertilized trees of Golden and Red Delicious, respectively. Accordingly, vulnerability thresholds representing embolism onset and full embolism were also shifted ~1.74 (P_12_) MPa and 0.7 (P_88_) MPa and 0.73 (P_12_) MPa and 0.23 (P_88_) MPa towards less negative values in fertilized trees of Golden and Red Delicious, respectively (Table 2; Fig. 1). Moreover, upon fertilization, Ψ_sc_ was shifted significantly ~0.7 MPa towards more negative leaf water potentials (Table 2).

### Stomatal conductance and cell osmotic parameters

Diurnal courses of *g*_s_ and Ψ_l_ of fertilized and control plants on an average summer day in 2015 followed similar patterns, but differed between cultivars (Supplementary Fig. S1). In both cultivars, *g*_s_ increased during the morning, and the highest values were reached around noon. In Golden Delicious, *g*_s_ then decreased steadily, and the reduction was faster in fertilized plants. In Red Delicious, *g*_s_ remained high until late afternoon and decreased at ~17.00 h CET. In both cultivars, we observed pre-dawn water potentials (Ψ_PD_) close to 0 MPa, and, following stomatal opening, a decrease in Ψ_l_ towards noon. In Golden Delicious, the lowest values were reached at ~16.00 h CET, and in Red Delicious at around midday. In Golden Delicious, significantly lower Ψ_lmin_ and higher *g*_smax_ were measured in fertilized (–3.04±0.07 MPa and 617±30 mmol m^–2^ s^–1^) compared with control trees (–2.73±0.11 MPa and 497±44 mmol m^–2^ s^–1^; Table 2). In Red Delicious, Ψ_lmin_ and *g*_smax_ ranged around –2.5 MPa and 555 mmol m^–2^ s^–1^, respectively.

In fertilized trees of Golden Delicious, turgor loss occurred at significantly less negative leaf water potentials (–2.65 MPa) compared with control trees (–2.97 MPa). A similar though not significant trend was observed for Ψ_osat_ (–2.39 MPa and –2.56 MPa; Table 2). In Red Delicious, Ψ_TLP_ and Ψ_osat_ were slightly higher than in Golden Delicious, but no significant differences between treatments were found (Table 2). In contrast, differences in *a*_ela_ between treatments were more pronounced (though not significant) in Red Delicious (1.5 times higher in fertilized plants). In both cultivars, the higher *a*_ela_ points to a lower cell wall elasticity in fertilized trees.

### Xylem and leaf anatomy

**Fig. 2.**
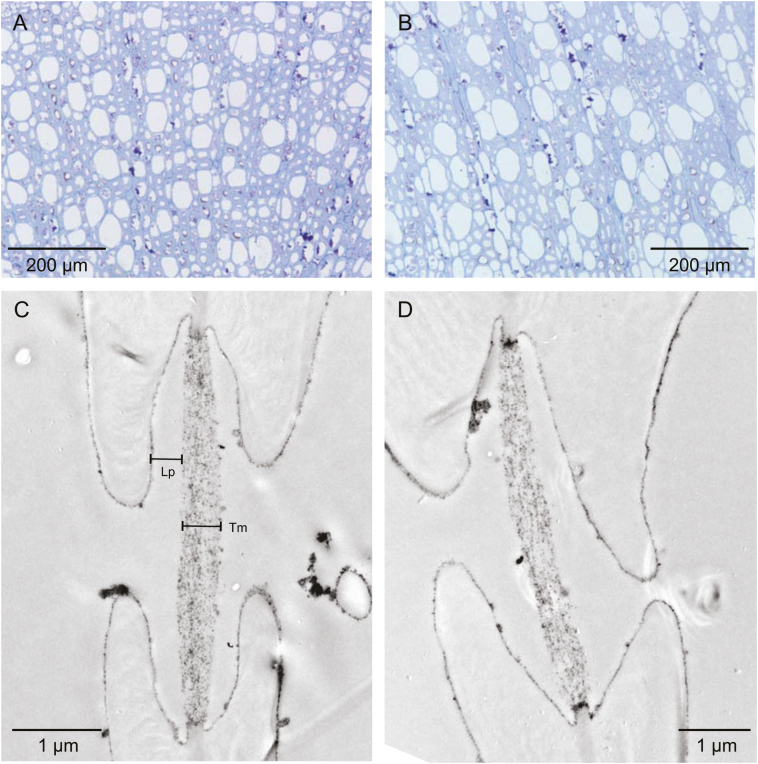
Transverse wood anatomical sections of control (A, C) and fertilized (B, D) trees of Golden Delicious. The light microscope images (A, B) show variation in conduit diameter and cell wall reinforcement, and the TEM images (C, D) demonstrate pit membrane thickness (*T*_m_) and pit chamber depth (*L*_p_).

**Fig. 3.**
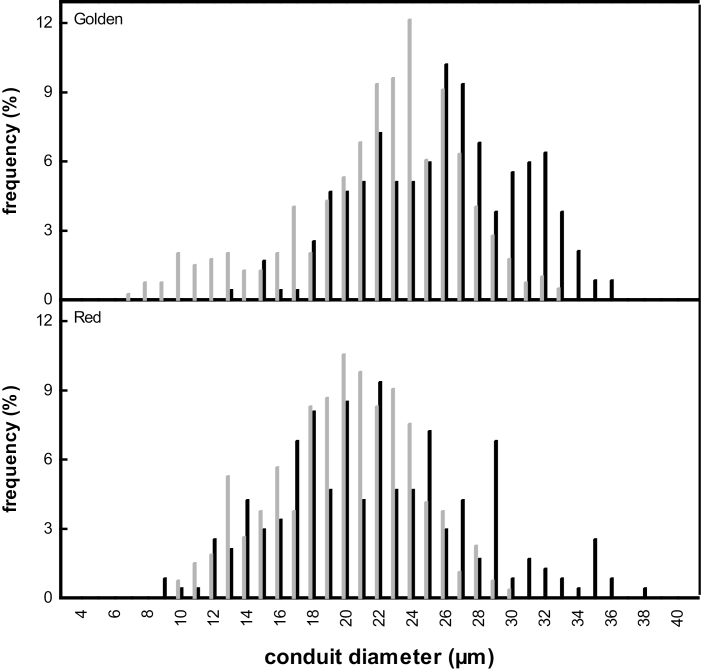
Frequency distribution of conduit diameters in the xylem of control (grey bars) and fertilized (black bars) of trees of Golden Delicious (Golden) and Red Delicous (Red) apple cultivars.

Larger *d*_mean_, *d*_h_, and *d*_max_ were found in fertilized trees (Table 2; Fig. 2), corresponding to higher hydraulic efficiency. Differences were not significant due to high variation in fertilized trees. For instance, *d*_mean_ ranged from 20 µm to 29 µm in fertilized plants of Golden Delicious, and from 16 µm to 27 µm in Red Delicious, while in control plants values ranged from 21 µm to 25 µm, and from 17 µm to 21 µm, respectively (data not shown). Higher values of fertilized trees were caused by higher fractions of wide conduits and, particularly in Golden Delicious, by a higher fraction of narrow conduits in control plants (Fig. 3). In both cultivars, (*t*/*b*)_h_^2^ was reduced from ~5.6 in control trees to 4.0 in fertilized trees (Table 2). Due to the high variation in fertilized trees of Red Delicious (values ranging from 1.7 to 6.3), differences were only significant in Golden Delicious.

Intervessel pit membranes appeared as electron-transparent structures with dark particles after OsO_4_ treatment. The thickness was more or less homogeneous across the membrane, but typically highest in the centre near the pit aperture. In Golden and Red Delicious, *T*_m_ tended to be lower in fertilized trees (415±17 nm and 320±40 nm) compared with control trees (459±50nm and 382±31 nm). *L*_p_ increased upon fertilization, but differences were only significant in Golden Delicious.

Regarding leaf anatomy, *l* was significantly higher in fertilized plants of Golden Delicious (17.61±0.47 µm) than in controls (15.03±0.59 µm), while in Red Delicious the opposite was observed (15.33±0.36 µm versus 17.27±0.51 µm; Table 2). SD was ~260 stomata mm^–2^ regardless of cultivar and treatment.

## Discussion

A well-balanced fertilization management is of utmost importance in apple orcharding to obtain high fruit quality and quantity. It also positively influences tree growth, although to what extent strongly depends on plant age, cultivar, rootstock, soil, and other management practices (Jackson, 2003; Carranca *et al.*, 2018). In our study, effects on growth were relatively low which might be related to the cultivars’ growth patterns as well as to growing conditions. Both cultivars were grafted on the dwarfing rootstock M9, which supports weak to moderate vigour, and trees were carefully pruned. Furthermore, Red Delicious plants were spur-types, which are characterized by a particularly compact growth with closely spaced fruit spurs and not particularly dominant trunks (Jackson, 2003). This might explain the even weaker response to fertilization in Red Delicious than in Golden Delicious (Table 1). A weak or lack of response to fertilization might also be attributed to a sufficiently high N content in control trees. The focus of this study was on potential consequences of fertilization on growth and hydraulic traits; thus, we did not consider nutrient uptake or tissue nutrient concentrations *per se*. However, partially highly significant differences between treatments were observed (see below) and, based on the knowledge that young fruit trees in particular strongly depend on sufficient nutrient supply for growth (Carranca *et al.*, 2018), we assume that the observed weak growth was attributed to limited water supply. The study year 2015 was relatively warm and dry. Although no prolonged and threatening drought periods occurred due to frequent thunderstorms, conditions might have been limiting, especially for Red Delicious, which strongly depends on high water supply for optimal performance. Despite its limited impact on growth, fertilization affected tree hydraulics considerably.

### Hydraulic efficiency of the branch xylem

In both cultivars, specific hydraulic conductivity (*k*_s_) of the branch xylem was significantly higher in fertilized than in control trees (Table 2). This was related to overall larger and most probably longer conduit diameters (Liu *et al.*, 2017), higher fractions of large diameter conduits, and, in the case of Golden Delicious, lower fractions of small diameter conduits compared with control trees (Table 2; Figs 2, 3). The also observed high variation of *k*_s_ in fertilized trees is related to a high variation in conduit diameters. Both increases as well as decreases in *k*_s_ have been reported upon fertilization (e.g. Clearwater and Meinzer, 2001; Bucci *et al.*, 2006; Hacke *et al.*, 2010; Plavcová and Hacke, 2012, 2013; Villagra *et al.*, 2013; Medeiros *et al.*, 2016). However, a comparison between studies is difficult due to different experimental setups (from selective fertilization under controlled conditions to manipulation of nutrient availablity of naturally nutrient deficient soils), plant age (from rooted cuttings or seedlings to mature trees), fertilizer (single nutrient to multinutrient fertilizers), fertilizing treatment (from moderate to excessive supply of fertilizer), and growing conditions (e.g. different light or water regimes).

Since ~58% of the total hydraulic resistivity of xylem is attributed to bordered pits between adjacent conduits (Choat *et al.*, 2008), pit properties, such as pit membrane porosity and thickness or pit aperture/pit membrane area, play a key role for flow resistance (Wheeler *et al.*, 2005; Choat *et al.*, 2008; Lens *et al.*, 2011). In the present study, the more conductive fertilized trees showed thinner pit membranes with deeper pit chambers, with the effects more pronounced in Golden Delicious (Table 2; Fig. 2). These observations are in line with Lens *et al.* (2011), who reported hydraulic conductivity to be negatively correlated with pit membrane thickness (*T*_m_) and positively with pit chamber depth (*L*_p_). A functional explanation for these correlations is still unclear. It has been assumed that thinner pit membranes provide less hydraulic resistance than thicker ones, possibly due to higher pit membrane porosity (e.g. Jansen *et al.*, 2009), and that deeper chambers increased embolism resistance (Lens *et al.*, 2011). Alternatively, pore sizes could be determined by how much a pit membrane is shrunken, which is not necessarily related to pit membrane thickness but could be affected by dehydration and frost (Li *et al.*, 2016; Schenk *et al.*, 2017; Zhang *et al.*, 2017).

### Hydraulic conductance of the root system

Although it is known that N supply often leads to a shift in biomass allocation from root towards stem and leaf tissues (e.g. Ewers *et al.*, 2000; Cooke *et al.*, 2005; Wang *et al.*, 2016), little is known about root hydraulics under different nutrient supply, and results are contradictory. In some studies, a reduction in the hydraulic conductance of roots (*K*_R_) of fertilized trees has been observed (e.g. Ewers *et al.*, 2000), although the decrease was often more pronounced under moderate levels and diminished with higher levels of N supply, or differences were only found under full sunlight but not under shaded conditions (Luis *et al.*, 2010; Wang *et al.*, 2016). In contrast, Trubat *et al.* (2006, 2012) reported a higher *K*_R_ for *Pistacia lentiscus* seedlings with sufficient N supply compared with plants under N deficiency conditions. Our study cultivars showed contrasting responses: while in Red Delicious, a slight reduction in *K*_R_ was observed upon fertilization, we found a strong increase in Golden Delicious (Table 2). At first glance, this seems interesting as both cultivars are grown on the same rootstock. However, recent studies have shown that scions of grafted plants have large influences on root biomass via different levels of endogenous abscisic acid (e.g. Chen *et al.*, 2002; McAdam *et al.*, 2016). Accordingly, the higher *K*_R_ of fertilized Golden Delicious plants might be related to a larger amount of fine roots and the consequently lower resistance in radial pathways (Tyree and Zimmermann, 2002).

### Embolism resistance

The ability to prevent xylem embolism is one crucial factor for a plant’s drought resistance. In both study cultivars, water potentials inducing 12, 50, and 88% loss of hydraulic conductivity (P_12_, P_50_, and P_88_) were significantly less negative in fertilized trees (Table 2; Fig. 1). Again, differences were more pronounced in Golden Delicious. This outcome is in line with most studies dealing with the effects of fertilization on embolism resistance in trees. Nevertheless, contradictory findings have been reported for different organs (Ewers *et al.*, 2000) and different nutrients (see the Introduction and Supplementary Table S1).

The decrease in embolism resistance was related to lower cell wall reinforcement [(*t*/*b*)_h_^2^; Table 2]. Positive correlations between embolism resistance and (*t*/*b*)_h_^2^ have been reported in several studies and on different taxonomical levels (Hacke *et al.*, 2001; Lens *et al.*, 2011). In some other fertilization studies, wood density has been positively correlated with embolism resistance (Hacke *et al.*, 2010; Plavcová and Hacke, 2012; Plavcová *et al.*, 2013). However, as wood density is influenced more by the thickness-to-span of fibres than conduits (Ziemińska *et al.*, 2013), this correlation is weaker and should not be used as a single determinant of embolism resistance (Cochard *et al.*, 2008; Lens *et al.*, 2011). Besides cell wall reinforcement, embolism resistance is also known to be coupled to pit characteristics, especially to *T*_m_ (Jansen *et al.*, 2009; Lens *et al.*, 2011; Scholz *et al.*, 2013; Li *et al.*, 2016), although the underlying mechanisms are largely unknown. It has been assumed that thicker pit membranes are less likely to have larger pores, thus increasing air-seeding pressure. However, this has not been proven yet and, as already discussed in context with hydraulic efficiency, porosity may largely be determined by the microfibril arrangement and be independent from *T*_m_ (Li *et al.*, 2016). Also, pit membranes are three-dimensional structures and thus air-seeding is rather determined by the most narrow pore throat within the largest pore pathway (Schenk *et al.*, 2017). Similar to Lens *et al.* (2011), Scholz *et al.* (2013), and Li *et al.* (2016), in our study embolism resistance was positively correlated with *T*_m_ and negatively with *L*_p_ (Table 2). Thereby, with values ranging from 320 nm to 459 nm, *T*_m_ was relatively high compared with other temperate tree species. Li *et al.* (2016), for instance, reported a mean *T*_m_ of 211 nm for a total of 49 temperate woody species, and Lens *et al.* (2011) observed a mean of 263 nm for seven *Acer* species. The high *T*_m_ was in accordance with the relatively negative P_50_ values found in our study cultivars. However, vulnerability thresholds to drought-induced embolism are only one aspect and by themselves not sufficient to characterize a plant’s drought tolerance. Another key trait is stomatal regulation as it determines whether a plant tends to a rather isohydric (sensitive stomatal control) or anisohydric (tolerating negative Ψ_l_) behaviour.

### Stomatal behaviour and drought tolerance of leaves

Individual plants can adjust their hydraulic behaviour with respect to water supply (e.g. Franks *et al.*, 2007; Domec and Johnson, 2012; Zhang *et al.*, 2012). We assume that this also applies to nutrient supply because in fertilized plants both onset and full stomatal closure occurred at more negative Ψ_l_ than in control trees (Table 2; Fig. 1). Unless low Ψ_l_ is avoided by a sufficiently high *k*_l_ and/or down-regulation of *g*_s_, late stomatal closure (Ψ_sc_) poses considerable risk of runaway embolism (Tyree and Sperry, 1988). In Red Delicious, neither Ψ_lmin_ nor *g*_smax_ differed between treatments (Table 2). In Golden Delicious, however, *g*_smax_ of fertilized trees was significantly higher, which might be related to significantly higher stomatal pore length (*l*) for similar stomatal density (SD) values (Table 2). A positive correlation between *l* and *g*_s_ has also been reported by Aasamaa *et al.* (2001) for various temperate deciduous trees, although higher *g*_smax_ is generally achieved with larger numbers of smaller but faster stomata (e.g. Franks *et al.*, 2009; Drake *et al.*, 2013; Elliott-Kingston *et al.*, 2016; Sack and Buckley, 2016). The increase in *g*_s_ resulted in significantly lower Ψ_lmin_ (Table 2). Ψ_lmin_ of fertilized trees was already in the range of embolism onset (Table 2), which might also explain the sharp drop in *g*_s_ in the afternoon (Supplemenatry Fig. S1). Although Ψ_l_ of transpiring leaves was probably more negative than actual water potential of the branch xylem, this cultivar follows a risky hydraulic strategy because of late stomatal closure (Supplementary Fig. S2).

Due to late stomatal closure, fertilized trees of both cultivars also operated at a higher risk of turgor loss (Supplementary Fig. S2). That was especially the case in Golden Delicious, as the turgor loss point (TLP) occurred at significantly less negative water potential in fertilized plants. The osmotic water potential at full saturation (Ψ_osat_) and cell wall elasticity (*a*_ela_) of this cultivar were high (Table 2), thus pointing to a reduction in drought tolerance of leaves upon fertilization (Sack *et al.*, 2003; Lenz *et al.*, 2006; Bartlett *et al.*, 2012).

### Fertilization or water supply: what matters most?

In the present study, considerable adjustments in tree hydraulics of two high-yield apple cultivars have been observed upon fertilization. Based on changes in xylem and pit anatomical traits, hydraulic efficiency was increased at the cost of embolism resistance in fertilized trees. Due to the parallel shift towards less negative Ψ_sc_ and the reduced drought tolerance of leaves (Golden Delicious), fertilized trees thus operated at the edge of hydraulic failure. A similar and remarkable risky hydraulic behaviour has also been observed in trees growing in commercial apple orchards (Beikircher *et al.*, 2013), where trees were irrigated daily. Thus, we suppose that optimal water supply might further increase the effect of fertilization. Nevertheless, given the pronounced cultivar-specific differences in various hydraulic and anatomical parameters reported by Beikircher *et al.* (2013), structure–functional changes in some cultivars might be limited by reduced water availability. Accordingly, we assume that in Red Delicious additional irrigation might have resulted in more pronounced differences in xylem and pit anatomy, maximum stomatal conductance, as well as leaf hydraulic parameters. In this context, the outcome of the present study is also of relevance for future orchard management. In view of altered precipitation patterns and reduced water availability due to climate change, careful selection of apple cultivars is required to ensure high productivity even under drier conditions.

## Supplementary data

Supplementary data are available at *JXB* online.

Table S1. Synthesis of studies investigating the effect of fertilization on the xylem embolism resistance of woody plants.

Table S2. List of parameters measured with acronyms, definition, and units.

Fig. S1. (A, B) Diurnal course of stomatal conductance and leaf water potentials of control and fertilized Golden Delicious and Red Delicious. (C) Air temperature, relative air humidity, and global radiation on the measurement day. Means ± SE.

Fig. S2. Sequence of characteristic hydraulic parameters during dehydration of control and fertilized plants of Golden Delicious and Red Delicious.

Supplementary Figure S1-S2

Supplementary Table S1-S2

## References

[CIT0001] Aasamaa K, Sober A, Rahi M 2001 Leaf anatomical characteristics associated with shoot hydraulic conductance, stomatal conductance and stomatal sensitivity to changes of leaf water status in temperate deciduous trees. Australian Journal of Plant Physiology 28, 765–774.

[CIT0002] Bartlett MK, Scoffoni C, Sack L 2012 The determinants of leaf turgor loss point and prediction of drought tolerance of species and biomes: a global meta-analysis. Ecology Letters 15, 393–405. 22435987 10.1111/j.1461-0248.2012.01751.x

[CIT0003] Beikircher B, Ameglio T, Cochard H, Mayr S 2010 Limitation of the Cavitron technique by conifer pit aspiration. Journal of Experimental Botany 61, 3385–3393. 20551085 10.1093/jxb/erq159

[CIT0004] Beikircher B, De Cesare C, Mayr S 2013 Hydraulics of high-yield orchard trees: a case study of three *Malus domestica* cultivars. Tree Physiology 33, 1296–1307. 24319028 10.1093/treephys/tpt096

[CIT0005] Beikircher B, Mayr S 2008 The hydraulic architecture of *Juniperus communis* L. ssp. *communis*: shrubs and trees compared. Plant, Cell & Environment 31, 1545–1556. 10.1111/j.1365-3040.2008.01860.x 18657057

[CIT0006] Beikircher B, Mayr S 2009 Intraspecific differences in drought tolerance and acclimation in hydraulics of *Ligustrum vulgare* and *Viburnum lantana*. Tree Physiology 29, 765–775. 19364707 10.1093/treephys/tpp018

[CIT0007] Beikircher B, Mayr S 2016 Avoidance of harvesting and sampling artefacts in hydraulic analyses: a protocol tested on *Malus domestica*. Tree Physiology 36, 797–803. 10.1093/treephys/tpv130 PMC4910940 26705311

[CIT0008] Bucci SJ, Scholz FG, Goldstein G, Meinzer FC, Franco AC, Campanello PI, Villalobos-Vega R, Bustamante M, Miralles-Wilhelm F 2006 Nutrient availability constrains the hydraulic architecture and water relations of savannah trees. Plant, Cell & Environment 29, 2153–2167. 10.1111/j.1365-3040.2006.01591.x 17081249

[CIT0009] Carranca C, Brunetto G, Tagliavini M 2018 Nitrogen nutrition of fruit trees to reconcile productivity and environmental concerns. Plants 7, 4. 10.3390/plants7010004 PMC5874593 29320450

[CIT0010] Chen G, Lips SH, Sagi M 2002 Biomass production, transpiration rate and endogenous abscisic acid levels in grafts of flacca and wild-type tomato (*Lycopersicon esculentum*). Functional Plant Biology 29, 1329–1335. 10.1071/PP01263 32688731

[CIT0011] Choat B, Cobb AR, Jansen S 2008 Structure and function of bordered pits: new discoveries and impacts on whole-plant hydraulic function. New Phytologist 177, 608–625. 18086228 10.1111/j.1469-8137.2007.02317.x

[CIT0012] Choat B, Drayton WM, Brodersen C, Matthews MA, Shackel KA, Wada H, McElrone AJ 2010 Measurement of vulnerability to water stress-induced cavitation in grapevine: a comparison of four techniques applied to a long-vesseled species. Plant, Cell & Environment 33, 1502–1512. 10.1111/j.1365-3040.2010.02160.x 20444217

[CIT0013] Clearwater MJ, Meinzer FC 2001 Relationships between hydraulic architecture and leaf photosynthetic capacity in nitrogen-fertilized *Eucalyptus grandis* trees. Tree Physiology 21, 683–690. 11446997 10.1093/treephys/21.10.683

[CIT0014] Cochard H 2002 A technique for measuring xylem hydraulic conductance under high negative pressures. Plant, Cell & Environment 25, 815–819.

[CIT0015] Cochard H, Barigah ST, Kleinhentz M, Eshel A 2008 Is xylem cavitation resistance a relevant criterion for screening drought resistance among *Prunus* species? Journal of Plant Physiology 165, 976–982. 17997190 10.1016/j.jplph.2007.07.020

[CIT0016] Cochard H, Damour G, Bodet C, Tharwat I, Poirier M, Ameglio T 2005 Evaluation of a new centrifuge technique for rapid generation of xylem vulnerability curves. Physiologia Plantarum 124, 410–418.

[CIT0017] Cochard H, Herbette S, Barigah T, Badel E, Ennajeh M, Vilagrosa A 2010 Does sample length influence the shape of xylem embolism vulnerability curves? A test with the Cavitron spinning technique. Plant, Cell & Environment 33, 1543–1552. 10.1111/j.1365-3040.2010.02163.x 20444214

[CIT0018] Cooke JE, Martin TA, Davis JM 2005 Short-term physiological and developmental responses to nitrogen availability in hybrid poplar. New Phytologist 167, 41–52. 15948828 10.1111/j.1469-8137.2005.01435.x

[CIT0019] Domec JC, Johnson DM 2012 Does homeostasis or disturbance of homeostasis in minimum leaf water potential explain the isohydric versus anisohydric behavior of *Vitis vinifera* L. cultivars? Tree Physiology 32, 245–248. 22427373 10.1093/treephys/tps013

[CIT0020] Domec JC, Palmroth S, Ward E, Maier CA, Thérézien M, Oren R 2009 Acclimation of leaf hydraulic conductance and stomatal conductance of *Pinus taeda* (loblolly pine) to long-term growth in elevated CO_2_ (free-air CO_2_ enrichment) and N-fertilization. Plant, Cell & Environment 32, 1500–1512. 10.1111/j.1365-3040.2009.02014.x 19558405

[CIT0021] Drake PL, Froend RH, Franks PJ 2013 Smaller, faster stomata: scaling of stomatal size, rate of response, and stomatal conductance. Journal of Experimental Botany 64, 495–505. 23264516 10.1093/jxb/ers347 PMC3542046

[CIT0022] Elliott-Kingston C, Haworth M, Yearsley JM, Batke SP, Lawson T, McElwain JC 2016 Does size matter? Atmospheric CO_2_ may be a stronger driver of stomatal closing rate than stomatal size in taxa that diversified under low CO_2_. Frontiers in Plant Science 7, 1253. 27605929 10.3389/fpls.2016.01253 PMC4996050

[CIT0023] Ewers BE, Oren R, Sperry JS 2000 Influence of nutrient versus water supply on hydraulic architecture and water balance in *Pinus taeda*. Plant, Cell & Environment 23, 1055–1066.

[CIT0024] Faustino LI, Bulfe NM, Pinazo MA, Monteoliva SE, Graciano C 2013 Dry weight partitioning and hydraulic traits in young *Pinus taeda* trees fertilized with nitrogen and phosphorus in a subtropical area. Tree Physiology 33, 241–251. 23355634 10.1093/treephys/tps129

[CIT0025] Faustino LI, Moretti AP, Graciano C 2015 Fertilization with urea, ammonium and nitrate produce different effects on growth, hydraulic traits and drought tolerance in *Pinus taeda* seedlings. Tree Physiology 35, 1062–1074. 26232784 10.1093/treephys/tpv068

[CIT0026] Franks PJ, Drake PL, Froend RH 2007 Anisohydric but isohydrodynamic: seasonally constant plant water potential gradient explained by a stomatal control mechanism incorporating variable plant hydraulic conductance. Plant, Cell & Environment 30, 19–30. 10.1111/j.1365-3040.2006.01600.x 17177873

[CIT0027] Franks PJ, Drake PL, Beerling DJ 2009 Plasticity in maximum stomatal conductance constrained by negative correlation between stomatal size and density: an analysis using *Eucalyptus globulus*. Plant, Cell & Environment 32, 1737–1748. 10.1111/j.1365-3040.2009.02031.x 19682293

[CIT0028] Ganthaler A, Mayr S 2015 Dwarf shrub hydraulics: two Vaccinium species (*Vaccinium myrtillus*, *Vaccinium vitis-idaea*) of the European Alps compared. Physiologia Plantarum 155, 424–434. 25677081 10.1111/ppl.12333 PMC4949559

[CIT0029] Hacke UG, Plavcová L, Almeida-Rodriguez A, King-Jones S, Zhou W, Cooke JE 2010 Influence of nitrogen fertilization on xylem traits and aquaporin expression in stems of hybrid poplar. Tree Physiology 30, 1016–1025. 20610665 10.1093/treephys/tpq058

[CIT0030] Hacke UG, Sperry JS, Pittermann J 2004 Analysis of circular bordered pit function II. Gymnosperm tracheids with torus-margo pit membranes. American Journal of Botany 91, 386–400. 21653394 10.3732/ajb.91.3.386

[CIT0031] Hacke UG, Sperry JS, Pockman WT, Davis SD, McCulloh KA 2001 Trends in wood density and structure are linked to prevention of xylem implosion by negative pressure. Oecologia 126, 457–461. 28547229 10.1007/s004420100628

[CIT0032] Harvey HP, Van Den Driessche R 1997 Nutrition, xylem cavitation and drought resistance in hybrid poplar. Tree Physiology 17, 647–654. 14759904 10.1093/treephys/17.10.647

[CIT0033] Harvey HP, Van Den Driessche R 1999 Nitrogen and potassium effects on xylem cavitation and water-use efficiency in poplars. Tree Physiology 19, 943–950. 12651306 10.1093/treephys/19.14.943

[CIT0034] Hubbard RM, Ryan MG, Stiller V, Sperry JS 2001 Stomatal conductance and photosynthesis vary linearly with plant hydraulic conductance in Ponderosa pine. Plant, Cell & Environment 24, 113–121.

[CIT0035] Jackson JE 2003 Biology of apples and pears. New York: Cambridge University Press.

[CIT0036] Jansen S, Choat B, Pletsers A 2009 Morphological variation of intervessel pit membranes and implications to xylem function in angiosperms. American Journal of Botany 96, 409–419. 21628196 10.3732/ajb.0800248

[CIT0037] Jansen S, Schenk HJ 2015 On the ascent of sap in the presence of bubbles. American Journal of Botany 102, 1561–1563. 26400778 10.3732/ajb.1500305

[CIT0038] Jones HG 1992 Plants and microclimate. A quantitative approach to environmental plant physiology. Cambridge: Cambridge University Press.

[CIT0039] Lens F, Sperry JS, Christman MA, Choat B, Rabaey D, Jansen S 2011 Testing hypotheses that link wood anatomy to cavitation resistance and hydraulic conductivity in the genus *Acer*. New Phytologist 190, 709–723. 21054413 10.1111/j.1469-8137.2010.03518.x

[CIT0040] Lens F, Tixier A, Cochard H, Sperry JS, Jansen S, Herbette S 2013 Embolism resistance as a key mechanism to understand adaptive plant strategies. Current Opinion in Plant Biology 16, 287–292. 23453076 10.1016/j.pbi.2013.02.005

[CIT0041] Lenz TI, Wright IJ, Westoby M 2006 Interrelations among pressure–volume curve traits across species and water availability gradients. Physiologia Plantarum 127, 423–433.

[CIT0042] Li S, Klepsch M, Jansen S, Schmitt M, Lens F, Karimi Z, Schuldt B, Espino S, Schenk HJ 2016 Intervessel pit membrane thickness as a key determinant of embolism resistance in angiosperm xylem. IAWA Journal 37, 152–171.

[CIT0043] Liu M, Pan R, Tyree MT 2017 Intra-specific relationship between vessel length and vessel diameter of four species with long-to-short species-average vessel lengths: further validation of the computation algorithm. Trees 32, 51–60.

[CIT0044] Luis VC, Llorca M, Chirino E, Hernández EI, Vilagrosa A 2010 Differences in morphology, gas exchange and root hydraulic conductance before planting in *Pinus canariensis* seedlings growing under different fertilization and light regimes. Trees 24, 1143–1150.

[CIT0045] McAdam SA, Brodribb TJ, Ross JJ 2016 Shoot-derived abscisic acid promotes root growth. Plant, Cell & Environment 39, 652–659. 10.1111/pce.12669 26514625

[CIT0046] Medeiros JS, Tomeo NJ, Hewins CR, Rosenthal DM 2016 Fast-growing *Acer rubrum* differs from slow-growing *Quercus alba* in leaf, xylem and hydraulic trait coordination responses to simulated acid rain. Tree Physiology 36, 1032–1044. 27231270 10.1093/treephys/tpw045

[CIT0047] Naor A, Girona J 2012 Apple. In: Steduto P, Hsiao TC, Fereres E, Raes D, eds. Crop yield response to water. Rome: FAO, 332–345.

[CIT0048] Nardini A, Salleo S, Jansen S 2011 More than just a vulnerable pipeline: xylem physiology in the light of ion-mediated regulation of plant water transport. Journal of Experimental Botany 62, 4701–4718. 21765173 10.1093/jxb/err208

[CIT0049] Oddo E, Inzerillo S, La Bella F, Grisafi F, Salleo S, Nardini A 2011 Short-term effects of potassium fertilization on the hydraulic conductance of *Laurus nobilis* L. Tree Physiology 31, 131–138. 21367746 10.1093/treephys/tpq115

[CIT0050] Pallardy SG 2008 Physiology of woody plants. London: Elsevier.

[CIT0051] Pammenter NW, Vander Willigen C 1998 A mathematical and statistical analysis of the curves illustrating vulnerability of xylem to cavitation. Tree Physiology 18, 589–593. 12651346 10.1093/treephys/18.8-9.589

[CIT0052] Plavcová L, Hacke UG 2012 Phenotypic and developmental plasticity of xylem in hybrid poplar saplings subjected to experimental drought, nitrogen fertilization, and shading. Journal of Experimental Botany 63, 6481–6491. 23095999 10.1093/jxb/ers303 PMC3504499

[CIT0053] Plavcová L, Hacke UG, Almeida-Rodriguez AM, Li E, Douglas CJ 2013 Gene expression patterns underlying changes in xylem structure and function in response to increased nitrogen availability in hybrid poplar. Plant, Cell & Environment 36, 186–199. 10.1111/j.1365-3040.2012.02566.x 22734437

[CIT0054] Sack L, Cowan PD, Jaikumar N, Holbrook NM 2003 The ‘hydrology’ of leaves: co-ordination of structure and function in temperate woody species. Plant, Cell & Environment 26, 1343–1356.

[CIT0055] Sack L, Buckley TN 2016 The developmental basis of stomatal density and flux. Plant Physiology 171, 2358–2363. 27268500 10.1104/pp.16.00476 PMC4972277

[CIT0056] Schenk HJ, Espino S, Romo DM, et al 2017 Xylem surfactants introduce a new element to the cohesion–tension theory. Plant Physiology 173, 1177–1196. 27927981 10.1104/pp.16.01039 PMC5291718

[CIT0057] Scholz A, Rabaey D, Stein A, Cochard H, Smets E, Jansen S 2013 The evolution and function of vessel and pit characters with respect to cavitation resistance across 10 *Prunus* species. Tree Physiology 33, 684–694. 23933827 10.1093/treephys/tpt050

[CIT0058] Sperry JS, Tyree MT 1988 Mechanism of water stress-induced xylem embolism. Plant Physiology 88, 581–587. 16666352 10.1104/pp.88.3.581 PMC1055628

[CIT0059] Torres-Ruiz JM, Cochard H, Mayr S, Beikircher B, Diaz-Espejo A, Rodriguez-Dominguez CM, Badel E, Fernández JE 2014 Vulnerability to cavitation in *Olea europaea* current-year shoots: further evidence of an open-vessel artifact associated with centrifuge and air-injection techniques. Physiologia Plantarum 152, 465–474. 24611594 10.1111/ppl.12185

[CIT0060] Trubat R, Cortina J, Vilagrosa A 2006 Plant morphology and root hydraulics are altered by nutrient deficiency in *Pistacia lentiscus* (L.). Trees 20, 334–339.

[CIT0061] Trubat R, Cortina J, Vilagrosa A 2012 Root architecture and hydraulic conductance in nutrient deprived *Pistacia lentiscus* L. seedlings. Oecologia 170, 899–908. 22717626 10.1007/s00442-012-2380-2

[CIT0062] Tyree MT, Ewers FW 1991 The hydraulic architecture of trees and other woody plants. New Phytologist 119, 345–360.

[CIT0063] Tyree MT, Hammel HT 1972 The measurement of the turgor pressure and the water relations of plants by the pressure-bomb technique. Journal of Experimental Botany 23, 267–282.

[CIT0064] Tyree MT, Patino S, Bennink J, Alexander J 1995 Dynamic measurements of root hydraulic conductance using a high-pressure flowmeter in the laboratory and field. Journal of Experimental Botany 46, 83–94.

[CIT0065] Tyree MT, Sperry JS 1988 Do woody plants operate near the point of catastrophic xylem dysfunction caused by dynamic water stress? Answers from a model. Plant Physiology 88, 574–580. 16666351 10.1104/pp.88.3.574 PMC1055627

[CIT0066] Tyree MT, Zimmermann MH 2002 Xylem structure and the ascent of sap. Berlin: Springer Verlag.

[CIT0067] Villagra M, Campanello PI, Montti L, Goldstein G 2013 Removal of nutrient limitations in forest gaps enhances growth rate and resistance to cavitation in subtropical canopy tree species differing in shade tolerance. Tree Physiology 33, 285–296. 23436182 10.1093/treephys/tpt003

[CIT0068] Wang AY, Wang M, Yang D, Song J, Zhang WW, Han SJ, Hao GY 2016 Responses of hydraulics at the whole-plant level to simulated nitrogen deposition of different levels in *Fraxinus mandshurica*. Tree Physiology 36, 1045–1055. 27259635 10.1093/treephys/tpw048

[CIT0069] Wheeler JK, Sperry JS, Hacke UG, Hoang N 2005 Inter-vessel pitting and cavitation in woody *Rosaceae* and other vesselled plants: a basis for a safety *versus* efficiency trade-off in xylem transport. Plant, Cell & Environment 28, 800–812.

[CIT0070] Zhang Y, Oren R, Kang S 2012 Spatiotemporal variation of crown-scale stomatal conductance in an arid *Vitis vinifera* L. cv. Merlot vineyard: direct effects of hydraulic properties and indirect effects of canopy leaf area. Tree Physiology 32, 262–279. 22157418 10.1093/treephys/tpr120

[CIT0071] Zhang Y, Klepsch M, Jansen S 2017 Bordered pits in xylem of vesselless angiosperms and their possible misinterpretation as perforation plates. Plant, Cell & Environment 40, 2133–2146. 10.1111/pce.13014 28667823

[CIT0072] Ziemińska K, Butler DW, Gleason SM, Wright IJ, Westoby M 2013 Fibre wall and lumen fractions drive wood density variation across 24 Australian angiosperms. AoB Plants 5, plt046.

