# Supplementary material for: Does fertilization explain the extraordinary hydraulic behaviour of apple trees?
Source: J Exp Bot. 2019 Feb 22;70(6):1915–25. doi: 10.1093/jxb/erz070 (PMC6436149; doi:10.1093/jxb/erz070)

Fig. S1: (A, B) Diurnal course of stomatal conductance (g_s; lines) and leaf water potentials (Ψ_l; circles) of control (gray) and fertilised (black) of Golden Delicious (Golden) and Red Delicious (Red). (C) Air temperature (solid line), relative air humidity (dashed line) and global radiation (grey area) on the measurement day. Means ± SE. Please note that lowest Ψ_l in this graph is not consistent with Ψ_{lmin} given in table 2, as for the calculation of latter the eight lowest values, independent of measurement time, were used (see material and method section).

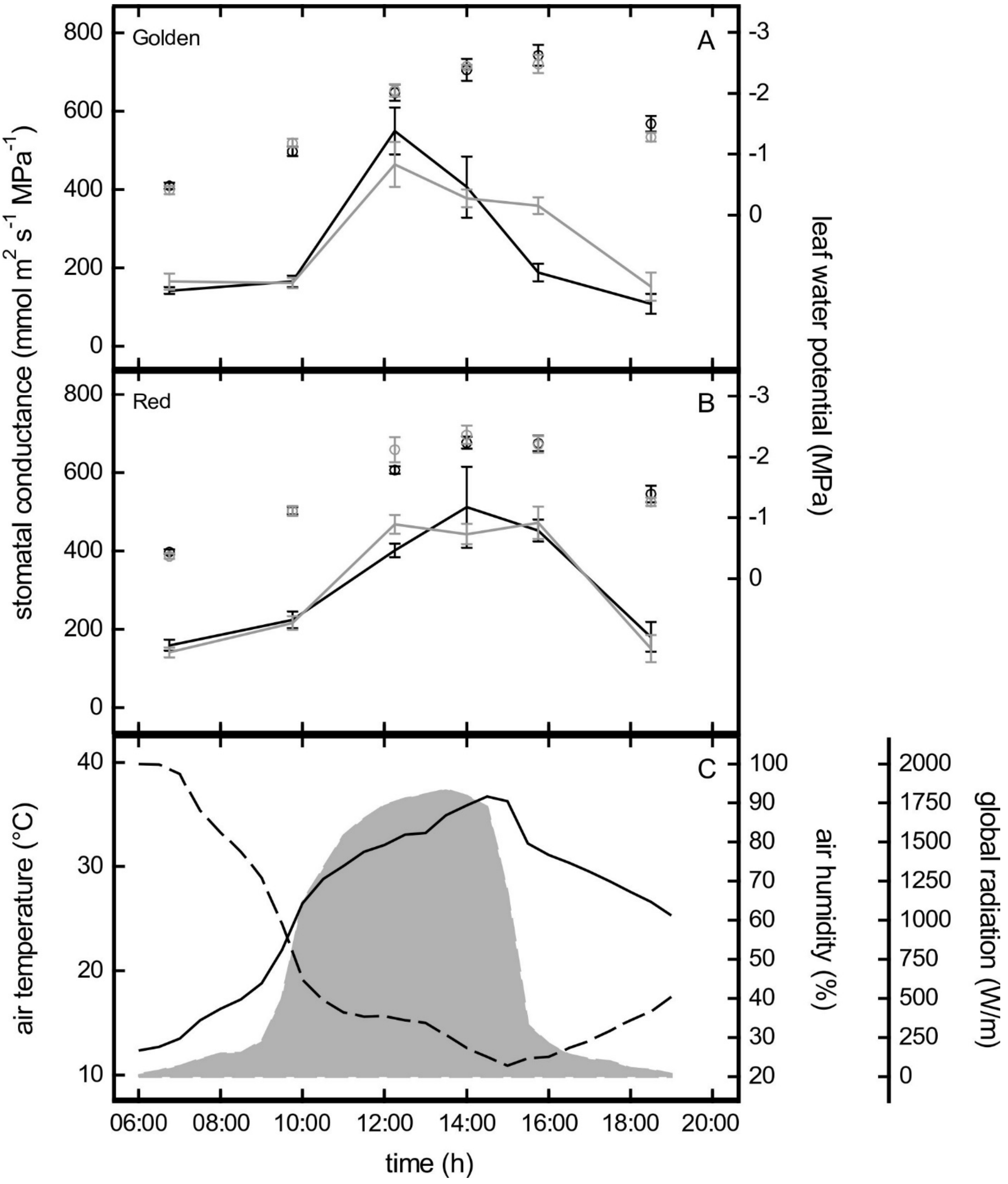

Fig. S2: Sequence of characteristic hydraulic parameters during dehydration of control and fertilised plants of Golden Delicious (Golden) and Red Delicious (Red). Bars show xylem pressures at 12, 50 and 88 % loss of hydraulic conductivity (P_{12}, P_{50}, P_{88}), vertical lines give water potentials at onset (dotted) and full (Ψ_{sc}; dashed) stomatal closure, symbols indicate turgor loss point (TLP; dark grey), predawn (Ψ_{PD}; open and light gray) and minimum leaf water potentials (Ψ_{lmin}; closed and medium gray) measured during a diurnal course. Please note that Ψ_{sc} and Ψ_{lmin} are measured on transpiring leaves and thus may not reflect stem water potential.

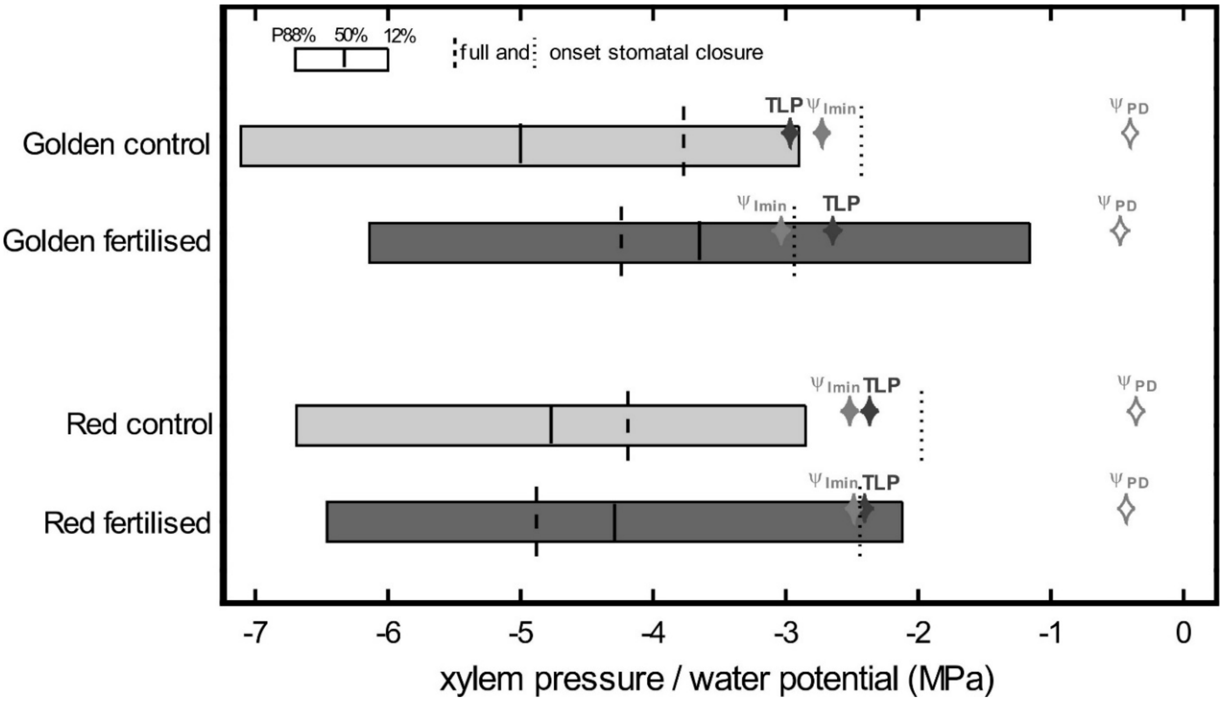

Supplement: Supplementary Figure S1-S2 [file erz070_suppl_supplementary_figure_s1-s2.pdf]
