# Supplementary material for: Does fertilization explain the extraordinary hydraulic behaviour of apple trees?
Source: J Exp Bot. 2019 Feb 22;70(6):1915–25. doi: 10.1093/jxb/erz070 (PMC6436149; doi:10.1093/jxb/erz070)
Supplement: Supplementary Table S1-S2 [file erz070_suppl_supplementary_table_s1-s2.pdf]

Table S1: Synthesis of studies investigating the effect of fertilisation on the xylem embolism resistance of woody plants

species	organ	measurement method	nutrient	response	reference
hybrid poplar	tree tops of rooted cuttings	flow measurements	N<br>P	decrease ↓<br>increase ↑	Harvey 1997
hybrid poplar	stem segments of rooted cuttings	dye diffusion method	N<br>K	decrease ↓<br>no effect ↔	Harvey 1999
hybrid poplar	stem segments of rooted cuttings	centrifuge method	N-P-K (20-20-20) fertilizer	decrease ↓	Hacke 2010
hybrid poplar	stem segments of rooted cuttings	flow measurements	N-P-K (20-20-20) fertilizer	decrease ↓	Plavcova 2012
hybrid poplar	stem segments of rooted cuttings	centrifuge method	N-P-K (20-20-20) fertilizer	decrease ↓	Plavcova 2013
savannah trees	terminal branches of adult trees	flow measurements	N<br>N+P<br>P	increase ↑<br>increase ↑<br>no effect ↔	Bucci 2006
tropical seasonally dry forest trees	terminal branches of adult trees	flow measurements	N+P	increase ↑	Villagra 2013
Pinus taeda	branches<br>roots	centrifuge method	N+P+K+Ca+Mg	no effect ↔<br>increase ↑	Ewers 2000

Table S2: List of parameters measured with acronyms, definition and units

Acronym	Definition	Units
P	xylem pressure	MPa
P_{12} / P_{50} / P_{88}	xylem pressure at 12, 50 and 88 % loss of hydraulic conductivity	MPa
PLC	percentage loss of hydraulic conductivity	%
a	slope of the vulnerability curve	-
Ψ_l	leaf water potential	MPa
Ψ_{PD}	predawn leaf water potential	MPa
Ψ_{lmin}	minimum leaf water potential	MPa
pV	pressure-volume	-
DW, FW, TW	dry, fresh and turgid weight	g
WSD	relative water saturation deficiciency	%
Ψ_{osat}	osomotic water potential at full saturation	MPa
Ψ_{TLP}	water potential at turgor loss point	MPa
a_{ela}	cell wall elasticity	-
k_s, k_l	specific and leaf specific hydraulic conductivity	$cm^2\ s^{-1}\ MPa^{-1}$
K_R	hydraulic conductance of the root system	$kg\ s^{-1}\ MPa^{-1}$
g_s	stomatal conductance	$mmol\ m^{-2}\ s^{-1}$
gs_{max}	maximum operating stomatal conductance	$mmol\ m^{-2}\ s^{-1}$
Ψ_{sc}	water potential at stomatal closure	MPa
SD	stomatal density	n/mm^2
l	length of stomatal pore	µm
d_{mean}	mean conduit diameter	µm
d_h	hydraulic diameter	µm
$(t/b)_h^2$	conduit wall reinforcement	-
T_m	pit membrane thickness	nm
D_{pm}	pit membrane diameter	µm
L_p	depth of the pit chamber	nm
SLA	specific leaf area	$m^2\ kg^{-1}$
